# The New Cardiff Medical School

**Published:** 1913-04-19

**Authors:** 


### The New Cardiff Medical School.

### THE EFFECT OF DR. H. R. VACHELL'S RETIREMENT.

At the monthly meeting of the board of management of the King Edward VII.'s Hospital, Cardiff, the resignation of the senior honorary physician, Dr. Herbert Redwood Vachell, was received in accordance with the rules of the hospital regarding the age limit, and on the proposition of the Chairman, Major-General Lee, seconded by Colonel Bruce Vaughan (the chairman of the house committee), Dr. Vachell was unanimously appointed as honorary consulting physician to the hospital.

Eloquent tributes to Dr. Vachell's long and distinguished services were paid by the Chairman and Colonel Bruce Vaughan, the latter giving many interesting particulars relating to the twenty-eight years during which Dr. Vachell continued an active member of the staff. As a matter of fact, his association with the hospital practically covers a period of thirty-six years, as he was appointed house surgeon in 1877, when the hospital had only one resident. After a reference to Dr. Vachell's notable work as chairman of the medical board for the two preceding years, Colonel Vaughan reviewed the important advancements and additions for the proficiency and efficiency of the institution, and with all of which Dr. Vachell had been closely associated. "For instance, twenty-eight years ago there was no ophthalmic surgeon, no gynæcologist, no ear, nose and throat physician, and no pathologist, and these departments are now presided over by men who devote all their time to one limited field of work, which work has yielded some of the great triumphs of the profession."

Dr. Vachell's resignation had a special significance coming at this point in the history of the hospital, as twelve members of the board of management were elected at this same meeting to serve on the newly-constituted election committee, and the appointments to the vacancy caused through Dr. Vachell's retirement and to that arising through the death of the late Dr. Arthur Taylor will be made by this new authority. With special reference to this aspect of the question, Colonel Vaughan, continuing, said: "We must take care that the men who will be elected to succeed them are men of the highest qualifications, not only for the sake of the patients in the hospital, but because shortly the men you will elect today will be the men who may be called upon to be the teachers of our medical students and who may ultimately occupy Chairs in our medical school."

Colonel Vaughan's eloquent tribute has provoked a leading article in the *Western Mail* of the 10th inst., containing the following comments: "Within a short space of time Cardiff is to see the establishment of a full medical curriculum in connection with the college and hospital. In Cardiff we have the solitary medical school of Wales: The record of work already performed justifies the hope that the school may develop within a few years into one of the most important centres of medical education in the British Isles. . . . The standard of practice at Cardiff and the health of the people must be maintained: it lies in the hands of Cardiff citizens to secure the best they can for themselves. They can do so by equipping the medical school with the most modern means of investigating disease, and by staffing it with men who have a thorough grounding in modern methods."

